# Dosimetric benefits of daily treatment plan adaptation for prostate cancer stereotactic body radiotherapy

**DOI:** 10.1186/s13014-021-01872-9

**Published:** 2021-08-04

**Authors:** Miriam Eckl, Gustavo R. Sarria, Sandra Springer, Marvin Willam, Arne M. Ruder, Volker Steil, Michael Ehmann, Frederik Wenz, Jens Fleckenstein

**Affiliations:** 1 Department of Radiation Oncology, University Medical Centre Mannheim, University of Heidelberg, Theodor-Kutzer-Ufer 1-3, 68167 Mannheim, Germany; 2 Department of Radiation Oncology, University Hospital Bonn, University of Bonn, Bonn, Germany; 3 University Medical Center Freiburg, University of Freiburg, Freiburg im Breisgau, Germany

**Keywords:** Prostate stereotactic body radiotherapy, Adaptive radiotherapy, Adaptive treatment planning, Synthetic cone-beam CT

## Abstract

**Background:**

Hypofractionation is increasingly being applied in radiotherapy for prostate cancer, requiring higher accuracy of daily treatment deliveries than in conventional image-guided radiotherapy (IGRT). Different adaptive radiotherapy (ART) strategies were evaluated with regard to dosimetric benefits.

**Methods:**

Treatments plans for 32 patients were retrospectively generated and analyzed according to the PACE-C trial treatment scheme (40 Gy in 5 fractions). Using a previously trained cycle-generative adversarial network algorithm, synthetic CT (sCT) were generated out of five daily cone-beam CT. Dose calculation on sCT was performed for four different adaptation approaches: IGRT without adaptation, adaptation via segment aperture morphing (SAM) and segment weight optimization (ART1) or additional shape optimization (ART2) as well as a full re-optimization (ART3). Dose distributions were evaluated regarding dose-volume parameters and a penalty score.

**Results:**

Compared to the IGRT approach, the ART1, ART2 and ART3 approaches substantially reduced the V_37Gy_(bladder) and V_36Gy_(rectum) from a mean of 7.4cm^3^ and 2.0cm^3^ to (5.9cm^3^, 6.1cm^3^, 5.2cm^3^) as well as to (1.4cm^3^, 1.4cm^3^, 1.0cm^3^), respectively. Plan adaptation required on average 2.6 min for the ART1 approach and yielded doses to the rectum being insignificantly different from the ART2 approach. Based on an accumulation over the total patient collective, a penalty score revealed dosimetric violations reduced by 79.2%, 75.7% and 93.2% through adaptation.

**Conclusion:**

Treatment plan adaptation was demonstrated to adequately restore relevant dose criteria on a daily basis. While for SAM adaptation approaches dosimetric benefits were realized through ensuring sufficient target coverage, a full re-optimization mainly improved OAR sparing which helps to guide the decision of when to apply which adaptation strategy.

## Background

Through the widespread introduction of image-guidance in radiotherapy (IGRT) its delivery accuracy has continuously improved over the last decade [1]. Nevertheless, the acquired image information is still rarely being used to adapt the treatment sequence to the given patient morphology of the day. The standard image-guided treatment workflow for fractionated radiotherapy at a conventional medical linear accelerator (linac) may not adequately account for interfractional organ variations. Methods of adaptive radiotherapy (ART) can address this issue [2], for example by using daily cone-beam computed tomography (CBCT) images to adapt the initial treatment plan according to daily organ deformations, tissue motion or weight loss [3–5]. In this context, inferior CBCT image quality poses a major challenge for the clinical implementation of ART procedures at a conventional linac [6, 7]. Nowadays, this can mostly be overcome by deep learning algorithms which are trained a priori with specific multimodal image datasets (treatment planning CT (pCT) and CBCT) and are afterwards used to generate corrected synthetic CT (sCT) images. These sCT were proven to successfully allow for reasonably fast workflow steps like automated image segmentation and accurate dose calculation for different tumor sites [8–11].

Prostate cancer patients could benefit from ART-techniques since the dosimetric accuracy is compromised when applying one treatment plan over the entire treatment course [12, 13]. While it would be desirable to consider also intrafractional organ motion, ART can at least compensate for interfractional changes such as varying organ fillings and relative distances between the clinical target volume (CTV) and the organs at risk (OAR) which are predominant causes for dosimetric deviations [14, 15]. Not only because of the Covid-19 pandemic, ultra-hypofractionated stereotactic body radiotherapy (SBRT) for prostate cancer has been increasingly used recently [16]. Exhibiting promising outcomes in terms of toxicity and survival [17], clinical trials such as PACE-C [18], pHART3 [19], HYPO-RT-PC [20], hypo-FLAME [21] or HYPOSTAT [22] were found to be alternatives to the conventional normo-fractionated or moderately hypo-fractionated treatment schemes [23–25].

Generating a fully optimized treatment plan for every treatment fraction is typically too time-consuming after IGRT and while the patient is positioned on the treatment couch. Alternatively, treatment plan libraries have been proposed [26–28] which yet need an accurate estimate of possible organ deformations. A fast modification of the initial treatment sequence [29] or applying segment aperture morphing (SAM) based on the “morphology-of-the-day”, eventually followed by an adaptation of the weights (monitor units) and shapes of the initial segments [30–33], may already adequately solve the problem.

Aims of this work are, first, to determine the maximum overall benefit of daily treatment plan adaptation for hypofractionated prostate SBRT. Second, dose distributions created by different treatment plan adaptation procedures ranging from SAM to a full optimization are compared to the standard IGRT approach. Lastly, a recommendation under which circumstances a certain approach may be most appropriate based on image segmentation information is expressed.

## Methods

### Patient population

32 patients with primary prostate cancer of low or intermediate risk were included in this retrospective treatment planning study and analyzed after IRB approval (2018-836R-MA) obtained by the ethics committee II of the University of Heidelberg. The guidelines of the PACE-C trial for prostate SBRT [34] were chosen exemplary as a framework for this analysis. No further consent to participation in the SBRT study was necessary as all patients were treated with the established normo-fractionated regimen. Additional procedures compared to the standard radiation therapy treatment were not performed. Relevant patient demographics are displayed in Table 1. Patients in prone position or carrying endoprosthesis were excluded from the study. All methods and treatment procedures of the present study were carried out in accordance with regulations and relevant guidelines for imaging, structure delineation, treatment planning and treatment delivery in radiation therapy.

**Table 1:** Patient characteristics

Average age and range (years)	Pre-treatment PSA (ng/ml)	Gleason score	Tumor stage	Average target volume (cm^3^)	Body-mass-index (BMI)	Comorbidity
71.9 (48–88)	< 10: 20 patients 10–20: 12 patients	6 (3 + 3): 9 patients 7 (3 + 4): 23 patients	T1c: 15 patients T2a-T2c: 17 patients	65.3 ± 26.3 (prostate) 18.3 ± 5.5 (seminal vesicles)	26.6 ± 2.8	Diabetes: 6 Hypertension: 18 Cardiovascular condition: 9

### Image processing

All patients were initially treated with volumetric modulated arc therapy (VMAT) at a linac (VersaHD, Elekta AB, Stockholm, Sweden) with a cumulative dose of 75 Gy delivered in a normo-fractionated treatment scheme [35]. Daily kV-CBCT (XVI 5.0, Elekta AB) were acquired prior to each fraction with 120 kV tube voltage, 132mAs exposure time product, an axial length of 27.7 cm, a gantry velocity of 1 rpm and a slice thickness of 2 mm. After rigid image registration of the CBCT and the pCT in 3 positional and 3 rotational degrees of freedom (in XVI), the CBCT were retrospectively reconstructed in the reference system of the pCT with a slice thickness of 1 mm and imported into the treatment planning system (Monaco 5.17, Elekta AB).

Although being “strongly recommended” by the PACE-C guidelines [34], no fiducial markers were available for image guidance due to our clinical routine treatment setup. One scanner specific CT-number-to-electron-density calibration curve was applied to all pCT and sCT. According to the PACE-C treatment scheme (40 Gy in 5 fractions) [34], CBCT of every second treatment fraction were selected up to a total of 5 CBCT, starting with the first treatment fraction. The average time between the pCT and the first fraction CBCT was 4.2 ± 1.7 days. The validity of this approach is based on the assumption that anatomical changes in normo- and hypofractionated prostate RT are indistinguishable. With the help of an individually trained cycle-generative adversarial network (cycle-GAN) based pelvis sCT model in the research software ADMIRE (Elekta AB) all CBCT were converted into sCT, taking on average 30 s per dataset. Specifications of the GAN framework and the pelvis sCT model can be taken from previously published literature [10].

### SBRT treatment planning

An expert physician delineated prostate and seminal vesicles (SV) as well as the OAR bladder, rectum, bowel, bilateral femoral heads and penile bulb on pCT and sCT. According to PACE-C guidelines [34], the clinical target volume (CTV) was defined as the prostate plus the proximal 1 cm of the SV. The planning target volume (PTV) was generated by adding an isotropic margin of 4 mm to the CTV. Based on the dosimetric criteria of the PACE trial summarized in Table 2, a treatment planning template was generated and applied to the pCT of each patient with minor adjustments. A dual 360° arc VMAT delivery with a nominal beam acceleration potential of 10MV with flattening filter was used for all treatments. Dose calculation was Monte Carlo-based with a statistical uncertainty of 1% and a grid size of 2 mm.

**Table 2:** Dose criteria of the PACE-C trial [34]

Target volumes	Dose (Gy)	Volume
CTV	40	≥ 95% (allowed minor variations: 90%-94.9%)
PTV	36.25	≥ 95% (allowed minor variations: 90%-94.9%)
	34.4	98%
**Organs at risk**		
Rectum	36	< 2 cm^3^
	29	< 20%
	18.1	< 50%
Bladder	37	< 10 cm^3^
	18.1	< 40%
Bowel	18.1	< 5 cm^3^
	30	< 1 cm^3^
Femoral heads	14.5	< 5%
Penile bulb	29.5	< 50%

### Adaptive treatment planning approaches

Warm start optimization (WSO) is a common approach to efficiently adapt a previously generated treatment plan. By using information of the reference treatment plan such as fluence, beam angles, MLC shapes and/or weights the re-optimization includes a priori information in the optimization process to reduce the required calculation time. One possible realization of WSO is segment aperture morphing (SAM) which was previously established by Ahunbay et al. [32]. SAM performs a morphing of the MLC positions in beam’s eye view of every segment or control point according to the respective change between the reference and actual shape of the target volume. Afterwards, an additional modification of the segment weights (monitor units) of the original treatment plan segments or control points can be performed in order to reach the optimum conditions of a pre-defined goal dose distribution (e.g. reference on the pCT). Further improvements can be gained through a re-optimization of the MLC shapes of the SAM-adjusted treatment plan.

In total, four different treatment planning approaches were retrospectively generated on each sCT:

I. IGRT approach: In analogy to regular IGRT treatments, the reference treatment plan was copied unaltered to the registered sCT. The isocenter position (ISO) on the pCT was the center-of-mass of the CTV while the ISO position on the sCT was shifted based on the translational and rotational corrections for all dose calculations in all scenarios.
II. ART1 approach: SAM was performed on the original treatment plan, followed by a segment weights optimization. A limit of maximum iterations of 50, 20 and 5 was used for the segment weight, segment shape and the combined optimization, respectively, with an activated gradient control. According to PACE-C criteria the dose distribution was rescaled to a prescription dose of 40 Gy to cover 95% of the CTV in approaches (II)–(IV).
III. ART2 approach: Same as ART1 but a WSO of the segment weights as well as the segment shapes was performed.
IV. ART3 approach: No prior treatment sequence information was used. A full optimization with dose constraints identical to the ones on the pCT was performed.

All treatment planning steps were performed on a workstation with a dual core Intel Xeon E5-2687 W v4 3.0 GHz processor, 64 GB RAM and an Nvidia Quadro P6000 GPU. To further evaluate clinical feasibility, the calculation time of every treatment planning procedure was recorded.

### Dosimetric and statistical treatment plan analysis

Four different treatment plans on each of the five sCT for each of the 32 patients were generated (in total 4 × 5 × 32 = 640), adding up to a total of 160 treatment plans per IGRT, ART1, ART2 and ART3 approach. Mean dose-volume-histograms (DVH) with point-wise standard deviations (SD) over all patients and treatment fractions for the four treatment planning approaches were generated. Furthermore, the most relevant dose-volume constraints of the PACE guidelines [34] were compared. A treatment plan quality scoring system, as proposed by the ESTRO QUASIMODO group [36], was applied to characterize the overall benefit of plan adaptation per dose criterion and per treatment planning approach. The penalty score S was based on the percentage difference between an actual value of a dose-volume parameter M for a given dose distribution and the corresponding PACE-C based dose-volume constraint C (see Table 2). Only violations of dose criteria contributed to S which resulted in an optimal plan having a count of S = 0:

$${\text{S}}=\sum\limits_{{\text{n}}} {\left\{ {\begin{array}{*{20}c} {\left| {\frac{{{\text{M}}_{{\text{n}}} - {\text{C}}_{{\text{n}}} }}{{{\text{C}}_{{\text{n}}} }}} \right| \times 100{,}} & {{\text{if}}\;{\text{ criterion}}\;{\text{is}}\;{\text{exceeded}}} \\ 0{,} & {{\text{else}}} \\ \end{array} } \right.}$$

The summation index n refers in the following to one of two different summations: First, a summation over all 160 treatment plans per dose criterion (inter-modality comparison) and second, a summation over all four dose criteria per patient (inter-patient comparison). To test whether the results of a certain treatment adaptation approach were statistically different from the ones of a different treatment adaptation approach paired t-tests between the resulting dose-volume parameters were performed. A *p*-value of *p* < 0.05 was considered statistically significant.

With regard to possible consequences of daily organ deformations on the dose distribution, the overlap-volumes between the PTV and the OAR bladder V_PTV ∩ bladder_ as well as rectum V_PTV ∩ rectum_ were determined in (cm^3^). Pearson’s correlation coefficient was calculated between the overlap-volumes of the bladder and rectum, respectively, and the total penalty score S per treatment plan. Subsequently, in order to identify a threshold for potential OAR overdose by means of overlap-volumes, the following ratio was calculated for the IGRT, ART1, ART2 and ART3 approach: The amount of treatment plans with over-average overlap-volumes and coincident dose criteria V_37Gy_(bladder) > 9 cc or V_36Gy_(rectum) > 1.5 cc was divided by the amount of total treatment plans with V_37Gy_(bladder) > 9 cc or V_36Gy_(rectum) > 1.5 cc.

## Results

**Fig. 1:**
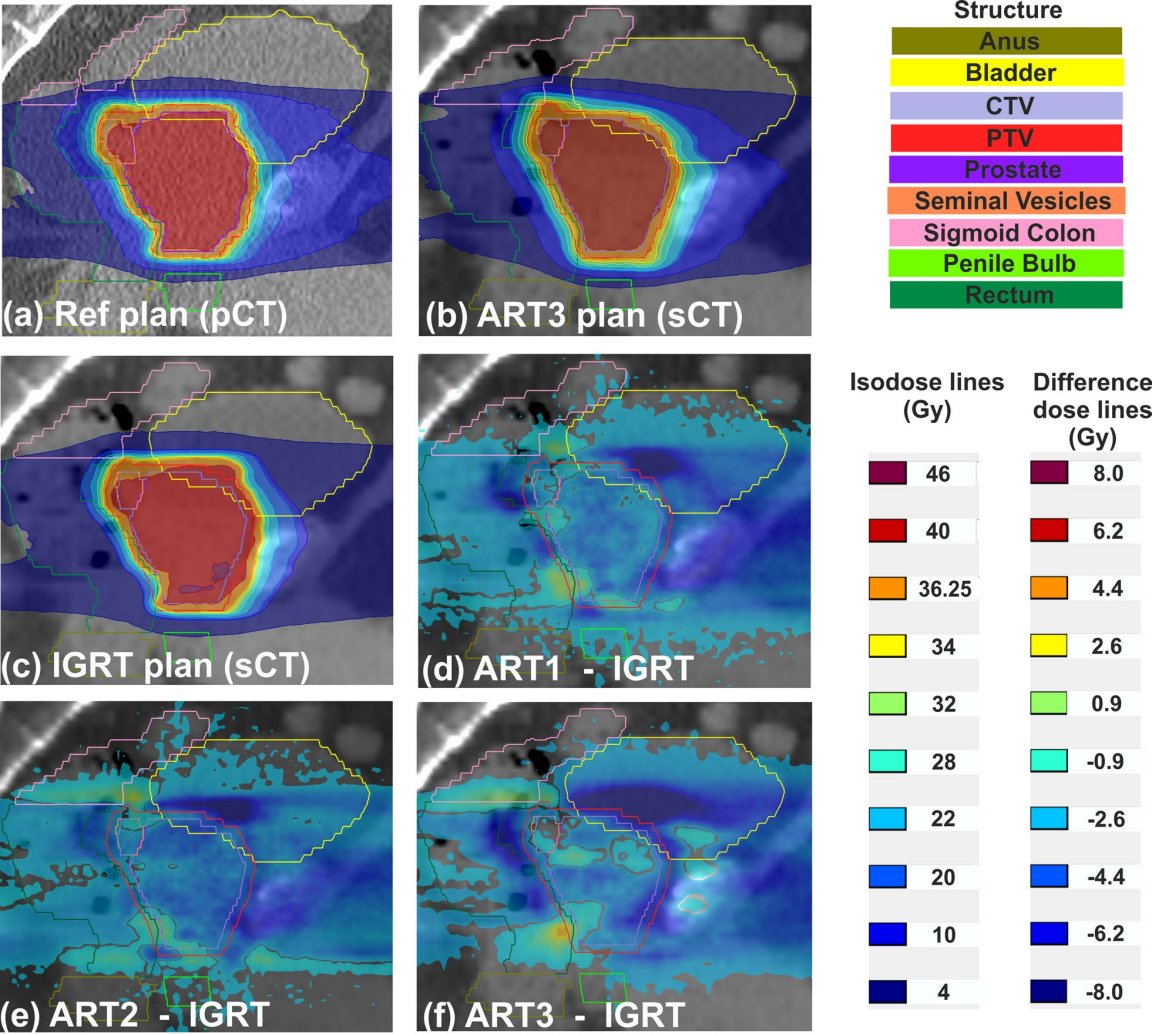
Exemplary sagittal dose distributions of the reference plan on the planning CT (**a**), of the ART3 plan on the synthetic CT (sCT) (**b**) and of the IGRT plan on the sCT (**c**). Dose difference maps between the IGRT approach and the three adaptive approaches on the sCT (**d**–**f**) revealed anatomical regions in the rectum, bladder and soft tissue with dose differences of up to 8 Gy. Prescription doses were D(CTV) = 40 Gy and D(PTV) = 36.25 Gy. A low dose threshold of 1% was used for the dose difference maps

Dose distributions of the reference plan (panel 1a) and of the ART3 and IGRT plans (panel 1a and 1b) as well as dose difference maps between the four treatment planning approaches in the sagittal isocenter plane (panels 1d,1e,1f) of one exemplary patient are shown in Fig. 1. In contrast to the pCT, a slightly deformed target structure and additional rectal flatus can be identified on the sCT. For the IGRT approach, normal tissue and OAR received doses higher than the PTV prescription dose of 36.25 Gy. These overdosages were reduced by up to 8 Gy (indicated by dark blue areas) by applying the ART1 or ART2 approach. The ART3 approach showed the highest dose sparing, especially along the PTV-bladder interface and in the cranial region of the rectum.

**Fig. 2:**
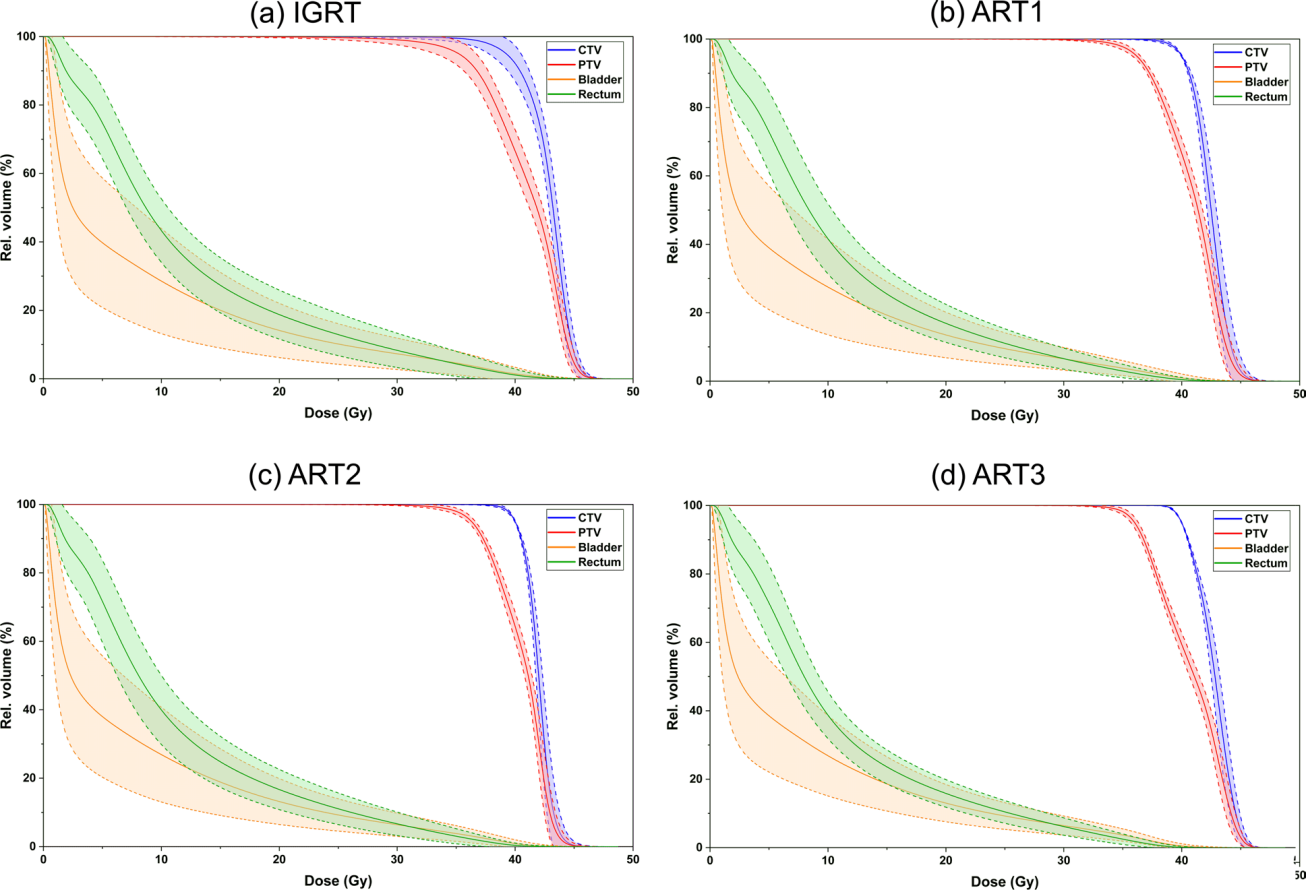
Mean dose-volume histogram of the CTV, PTV, bladder and rectum for the four adaptation approaches IGRT, ART1, ART2 and ART3. Light colored ribbons represent the point-wise standard deviation among a collective of 160 plans per treatment planning approach. Prescription doses were D(CTV) = 40 Gy and D(PTV) = 36.25 Gy

Figure 2 displays the averaged DVH over 160 treatment plans per adaptation approach including point-wise SD (light colored ribbons equate to one standard deviation) of the bladder, rectum, CTV and PTV. The IGRT approach showed the largest SD-ribbons for all four structures. The SD decreased with an increasing degree of adaptation, especially for the OAR. Compared to the ART1 and ART2 approach, the ART3 approach (panel 2d) had the narrowest SD-ribbons above 30 Gy for both OAR and near 45 Gy for the target volumes.

**Fig. 3:**
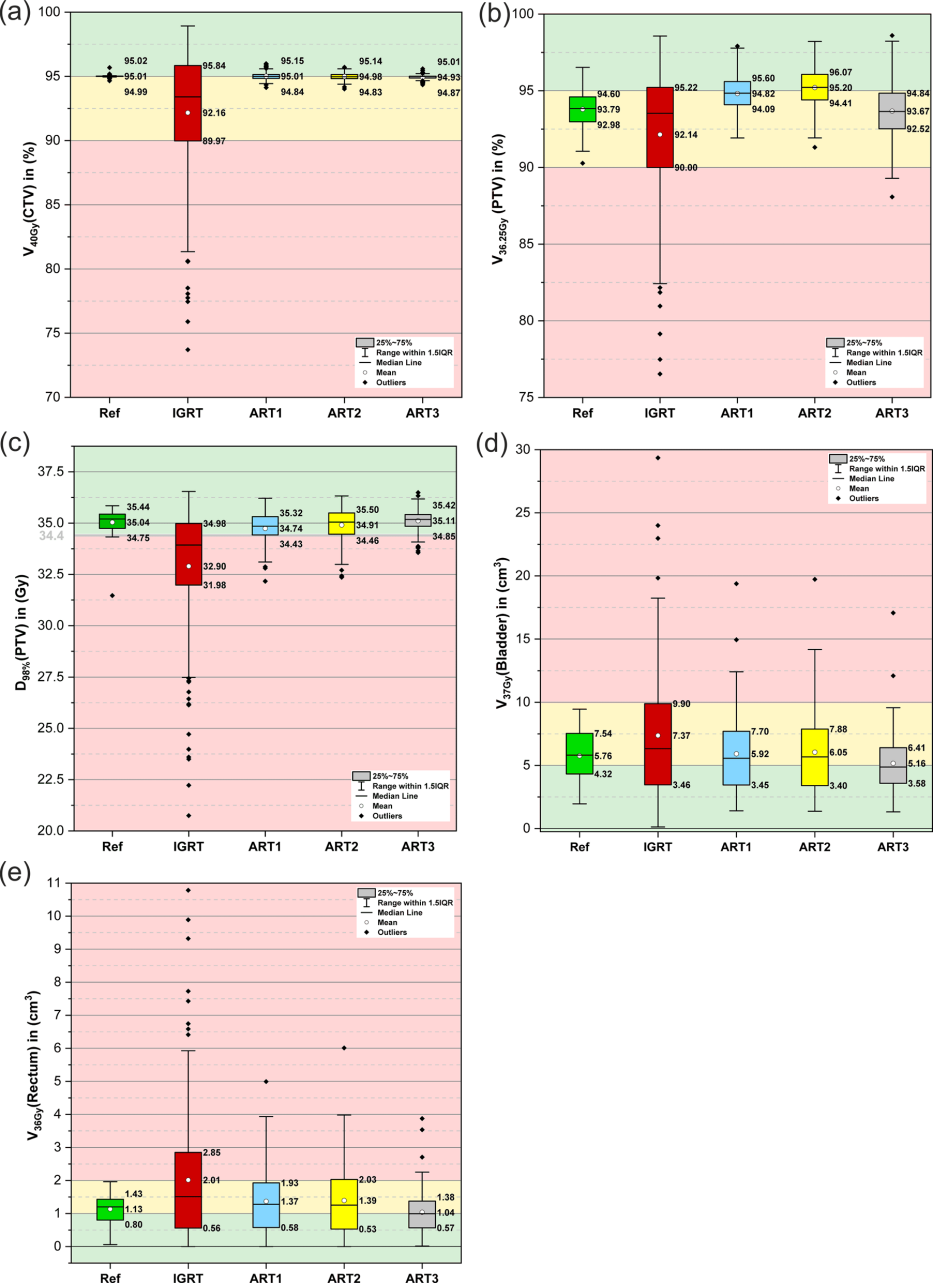
Boxplots of five dose-volume criteria for the CTV (**a**), PTV (**b**)–(**c**), bladder (**d**) and rectum (**e**) on the planning CT (Ref) and synthetic CT (IGRT, ART1, ART2 and ART3 approaches). Whiskers denote the data within 1.5 times of the interquartile range (IQR) based on 32 plans for the reference and 160 for the four adaptation approaches. Background colors indicate optimal (green), acceptable (yellow) and unacceptable (red) results according to the PACE-C treatment planning guideline

During the initial treatment plan optimization process, the most relevant (i.e. dose-shaping) dose-volume criteria were identified to be V_40Gy_(CTV), V_36.25 Gy_(PTV), D_98%_(PTV), V_37Gy_(bladder), V_36Gy_(rectum). Boxplots for these five dose-volume criteria are shown in Fig. 3 with respect to the reference dose distribution and the four adaptation approaches. With regard to CTV and PTV coverage (panel 3a and 3b), the IGRT approach yielded the largest variations with inter-quartile ranges (IQR) of 5.9% and 5.2% but stayed within the acceptable volume region of > 90%. Only the three re-optimization approaches (ART1-ART3) accomplished dose coverages in the optimum volume range close to 95%. Regarding the criterion D_98%_(PTV) (panel 3c), the mean value and large parts of the IQR of the IGRT approach were considerably below the goal value of 34.4 Gy while the ART3 approach achieved the highest value with 35.1 Gy with the smallest IQR of all three re-optimization approaches. Doses to OAR were highest for the IGRT approach with a mean of 7.4 cc and 2.0 cc for the V_37Gy_(bladder) and V_36Gy_(rectum) (panel 3d and 3e), respectively. Several outliers over 15 cc (bladder) and 3 cc (rectum) were noticeable for all three re-optimization approaches. However, for the bladder, all IQR of the re-optimization approaches were within the mandatory volume region below 8 cc and had mean values of < 6.1 cc. In general, the most protocol violations were found for V_36Gy_(rectum) > 2 cc where only the ART3 approach achieved a mean V_36Gy_ of < 1.3 cc.

The paired t-tests between the four adaptation approaches for the dose-volume criteria presented in Fig. 3 all yielded significant differences except for the correlation of V_36Gy_(rectum) between ART1 and ART2 approaches with *p* = 0.32. Due to the rescaling of D_95%_(CTV) to the prescription dose of 40 Gy for the adaptation approaches, V_40Gy_(CTV) was neither included in the statistical tests nor in the calculation of the penalty score S.

The results of the penalty score S summed over all 160 treatment plans per dose criterion as well as summed over all four dose criteria per patient/treatment plan is shown in Table 3. Regarding the first summation per dose criterion, the total penalty scores of the three re-optimization approaches were considerably lower than the related score of the IGRT approach (reduced by 79.2%, 75.7% and 93.2%). For the OAR dose criteria, the ART3 approach achieved the largest improvements compared to the IGRT approach with penalty scores reduced by 94.9% and 95.7% for bladder and rectum constraints, respectively. Averaged over the two PTV/OAR criteria, S was reduced by 86.3%/79.9%, 89.9%/76.0% and 75.0%/95.3% for the ART1, ART2 and ART3 approaches. For all four adaptation approaches, the violation of the criterion V_36Gy_(rectum) < 2 cc constituted the largest proportion of the total penalty score.

**Table 3:** Penalty score S per dose criterion and per patient/treatment plan for the reference plan and the four adaptation approaches

	Penalty score S per reference plan and adaptation approach				
Per dose criterion (summed over all patients)	Reference	IGRT	ART1	ART2	ART3
V_36.25 Gy_(PTV) ≥ 95%	47	544	93	66	262
D_98%_(PTV) ≥ 34.4 Gy	9	852	88	69	16
V_37Gy_(Bladder) < 10 cc	0	1800	292	333	92
V_36Gy_(Rectum) < 2 cc	0	5943	1424	1754	254
Total penalty score	56	9139	1897	2222	624

Per patient/treatment plan (summed over all dose criteria)	Reference	IGRT	ART1	ART2	ART3
Patient / CT	pCT	Mean ± SD (5 sCTs)	Mean ± SD (5 sCTs)	Mean ± SD (5 sCTs)	Mean ± SD (5 sCTs)
P1	2.0	14.2 ± 10.6	1.0 ± 0.5	0.3 ± 0.4	1.6 ± 0.9
P2	3.0	35.0 ± 36.5	3.4 ± 0.7	1.3 ± 0.4	2.3 ± 1.0
P3	1.6	67.7 ± 54.9	0.1 ± 0.2	5.9 ± 9.7	1.2 ± 0.8
P4	2.3	77.3 ± 51.0	27.7 ± 22.7	33.7 ± 25.7	21.0 ± 28.7
P5	1.0	24.9 ± 25.2	8.2 ± 15.7	5.0 ± 9.8	0.5 ± 0.6
P6	1.5	84.5 ± 104.9	22.5 ± 33.6	17.8 ± 33.5	2.9 ± 2.7
P7	4.1	82.5 ± 51.5	15.8 ± 20.3	19.5 ± 25	9.3 ± 14.0
P8	1.0	80.5 ± 64.4	17.7 ± 25.2	20.4 ± 21.4	1.7 ± 1.6
P9	1.8	9.9 ± 10.1	0.1 ± 0.2	0 ± 0.1	1.4 ± 0.1
P10	10.2	94.5 ± 85.3	20.8 ± 13.8	18.7 ± 8.5	4.8 ± 0.7
P11	1.2	18.9 ± 20.1	0.1 ± 0.1	0.1 ± 0.2	0.7 ± 0.6
P12	1.9	34.3 ± 23.7	1.5 ± 1.9	0.3 ± 0.6	2.0 ± 0.5
P13	0	25.8 ± 20.0	0.9 ± 1.8	0 ± 0	1.4 ± 1.1
P14	0.8	157.6 ± 218.6	39.8 ± 75.4	40.9 ± 77.8	19.9 ± 29.5
P15	2.0	58.4 ± 55.9	13.6 ± 21.9	17.7 ± 21.8	2.7 ± 1.1
P16	0	4.8 ± 4.4	0 ± 0.1	0 ± 0	0 ± 0.1
P17	1.2	38.3 ± 32.2	3.3 ± 6.3	8.5 ± 10.1	2.4 ± 0.7
P18	0.4	17.0 ± 14.5	0.8 ± 1.0	0.8 ± 1.0	1.1 ± 0.7
P19	2.5	58.3 ± 63.5	5.7 ± 5.5	6.1 ± 6.7	1.8 ± 1.3
P20	2.2	56.2 ± 24.0	7.1 ± 5.8	8.3 ± 5.6	1.9 ± 0.7
P21	0	7.3 ± 13.5	0.6 ± 0.6	0.3 ± 0.7	0 ± 0
P22	1.1	70.6 ± 21.4	24.7 ± 15.3	19.1 ± 14.0	6.6 ± 8.7
P23	2.2	46.0 ± 47.2	24.5 ± 32.4	17.0 ± 22.0	1.8 ± 0.6
P24	0	288.7 ± 115.4	53.8 ± 51.1	81.2 ± 76.3	24.6 ± 35.2
P25	5.0	57.7 ± 51.8	10.0 ± 9.2	18.2 ± 18.2	4.1 ± 0.6
P26	4.2	15.5 ± 14.9	2.3 ± 1.6	1.2 ± 1.7	1.8 ± 1.0
P27	0	71.5 ± 44.4	29.5 ± 23.6	41.1 ± 31.2	3.2 ± 3.7
P28	1.0	12.7 ± 25.4	0.6 ± 0.5	0.7 ± 1.1	0.4 ± 0.4
P29	0.5	210.2 ± 108.7	43.1 ± 21.9	59.9 ± 29.7	1.2 ± 1.3
P30	0.0	2.1 ± 3.1	0 ± 0	0 ± 0	0 ± 0
P31	1.2	0.9 ± 0.6	0 ± 0	0.1 ± 0.3	0.4 ± 0.4
P32	0.0	3.9 ± 7.7	0.1 ± 0.1	0.2 ± 0.4	0 ± 0
Mean penalty score	1.7 ± 2.0	57.1 ± 87.4	11.9 ± 25.7	13.9 ± 30.6	3.9 ± 11.8

With respect to the summation per patient/treatment plan, the largest mean penalty scores averaged over all five sCT were obtained for the IGRT approach with patient P14, P24 and P29 having S_mean_ = 157.6, S_mean_ = 288.7 and S_mean_ = 210.2. Together with patient P6, they showed the largest SD exceeding 100 and also led to comparably high values of S_mean_ and SD for the ART1 and ART2 approaches. For patient P27 and P29 only a full re-planning (ART3) was able to achieve scores of S_mean_ < 4 and SD < 4 whereas P4, 14 and P24 remained as the patients with most dosimetric violations having S_mean_ of at least 19.9 for the ART3 approach. Averaged over the entire patient collective, the mean penalty scores for the IGRT, ART1, ART2 and ART3 approaches were 57.1, 11.9, 13.9 and 3.9.

The overlap-volumes V_PTV ∩ bladder_ and V_PTV ∩ rectum_ were 7.2 ± 3.4 cc and 2.6 ± 1.3 cc averaged over all sCT. Pearson’s correlation coefficients between each of the two overlap-volumes and the total penalty score S per treatment plan for the planning approaches IGRT, ART1, ART2 and ART3 were 0.53, 0.49, 0.50, 0.46 (bladder) and 0.47, 0.52, 0.55, 0.43 (rectum), respectively.

For the IGRT approach, 68.5% and 69.1% of all treatment plans exhibiting high OAR doses of V_37Gy_(bladder) > 9 cc and V_36Gy_(rectum) > 1.5 cc coincided with an over-average overlap-volume for bladder and rectum, respectively. This proportion of correctly anticipated overdosage to bladder/rectum amounted to 100%/86.9%, 100%/90.1% and 100%/96.3% for the ART1, ART2 and ART3 approach.

Treatment plan generation times (dose calculation and segment adaptation, where applicable) below three minutes were obtained for the IGRT and ART1 approach with 2.5 ± 0.3 min and 2.6 ± 0.3 min. Due to the additional segment adaptation, the ART2 and ART3 scenarios required 12.1 ± 3.2 min and 19.4 ± 4.0 min to generate a treatment plan on the daily sCT. Since time is of crucial importance for daily ART, plan calculation was also performed with a grid size of 3 mm which led to total plan generation times of 0.8 ± 0.1 min, 0.9 ± 0.1 min, 4.8 ± 1.9 min and 6.7 ± 1.7 min for the IGRT, ART1, ART2 and ART3 approaches, respectively.

## Discussion

Previously published comparative studies on radiotherapy for prostate cancer with and without image guidance concluded the existence of dosimetric advantages of IGRT over non-IGRT [1, 37]. In this sense, the goal of this work was to evaluate whether further dosimetric benefits of different treatment plan adaptation approaches exist in comparison to the conventional IGRT approach. Although this study focused on the specific guidelines of the PACE-C trial, the presented outcomes emphasize the necessity of daily adapting the initial treatment plan and could be applied to other prostate cancer treatment schemes at conventional (non-MRI-based) linacs in an analogous manner.

For the IGRT approach large dose outliers as well as large overall penalty scores and comparably high SD in the DVH were obtained. This implies that the large variation of interfractional organ fillings and deformations cannot be compensated entirely by simply rotating and translating the patient as it has been previously reported [38, 39]. However, the presented IGRT approach did not achieve the precision as obtained by a workflow including fiducial markers. Furthermore, due to the more robust matching process, the time for image registration and manual delineation could have been reduced. In general, two reasons for treatment plan adaptation exist [40]: Adaptation of the treatment sequence to positional changes of the anatomy and/or to a deformed shape of the anatomy. In the first scenario, ART is feasible but not required and can be replaced by a marker-based IGRT which corrects for any translational and rotational degrees of freedom [41]. If, on the other hand, the anatomy at the time of treatment is noteworthy deformed, for example due to rectal or bladder filling, no translation or rotation will be sufficient to adequately compensate for these deficits and ART is a possible treatment approach.

But even in the presence of anatomical deformations at the time of image guidance, alternative methods to compensate for potential dosimetric deviations in relevant areas such as rectal spacers [14], rectal balloons [42] or strict bladder filling [43] exist which present an alternative to ART. While ART is typically more comfortable for the patient and, once the relevant technology is robustly available, is potentially easier to realize it might only be efficient for treatments with a low number of fractions or adaptation procedures. While combining motion mitigation techniques with SAM-based adaptation approaches like ART1 or ART2 could lead to dosimetric improvements and faster optimization times, a combination with a full re-optimization (ART3) may not generate further clinically relevant dosimetric benefits.

The evaluation of the proposed adaptation approaches revealed that an additional benefit over the IGRT dose distribution can already be achieved by modifying the weights of the original plan segments (ART1). This leads to a short treatment plan adaptation time of below three minutes and, due to the insignificant difference of the rectum dose-volume criterion compared to the ART2 approach, makes the ART1 approach the most favorable method of choice for an efficient use in daily clinical routine. An additional optimization of the initial segment shapes (ART2) could lead to improved target coverage and reduced hotspots, on the expenses of increasing the calculation time by a factor of 4.7 (5.3 for the 3 mm grid). These values can only be compared with some tolerance range to related research due to different plan delivery modes, hardware components, calculation settings and image resolutions. Plan generation times of other prostate and lymph node studies were reported as approximately 3 min (SAM plus SWO and dose calculation) [31], < 2 min (adaptive sequencer plus SWO including different shift methods) [44] and 11-119 s (different SAM methods at the MR linac with a 3 mm grid) [30] which compares well to our findings of 2.6 min for a grid of 2 mm for the ART1 approach (SAM plus WSO).

The ART3 approach yielded the highest treatment plan quality and lowest total penalty score since a full re-optimization can adapt best to the daily anatomy of the patient. A recent study also concluded that the most promising adaptive approaches for prostate treatments are re-planning procedures rather than MLC adjustments or pre-optimized plan libraries [2].

An explanation for the lowest obtained OAR doses of the ART3 approach can be found in the way the SBRT treatment planning template was built. It prioritized a sparing of the OARs over maximum target coverage, while ART1 and ART2 approaches aimed at a reconstruction of the initial target coverage [32]. Thus, a full re-optimization is only recommended for daily situations in which target structure deformations require large re-shaping of the MLC or OAR unexpectedly move into high dose areas. This outcome is in-line with findings of previous studies [45, 46] and, of course, dependent on the applied PTV margin [47].

The distribution of the mean penalty score S and the respective SD among the total population revealed The ART1 approach was already able to reduce the total S_mean_ of the IGRT approach from 57.1 to 11.9, including a reduction of the SD from 87.4 to 25.7. Most substantial improvements were concentrated rather on treatment plans of single patients (P14, P24 and P29) than scattered throughout the total treatment plan collective. Even the ART3 approach exhibited a few extraordinary criteria violations with a large SD > 25 for patients P4, P14 and P24 which is related to their comparably exceptional anatomy. All three patients showed over-average to very large mean V_PTV ∩ bladder_ and V_PTV ∩ rectum_ of (8.7 cc, 13.9 cc, 15.5 cc) and (4.9 cc, 3.0 cc, 4.1 cc), respectively. Thus, only a re-optimized treatment plan with altered dose constraints or an exclusion from the SBRT treatment would have solved this issue. The largest benefit of ART3 over the adaptive approaches of ART1 and ART2 for all five sCT was noticeable for patient P24. In this case, a high mean V_PTV ∩ rectum_ of 4.1 cc and comparable large daily CTV deformations in anterior direction of up to 0.60 cm due to rectal filling could not be accounted for by both SAM-based approaches ART1 and ART2. Despite its advantage over simpler pass/fail scoring systems, the presented penalty scoring has limitations by being more time consuming to calculate and prioritizing certain dose criteria like OAR violations over PTV violations. Although the penalty score S cannot replace the visual inspection of the spatial dose distribution, it offers a quick treatment plan evaluation at one glance. Noteworthy deviations from the reference score on the pCT can serve as an action trigger to re-evaluate the adapted plan and immediately detect exceptional dosimetric violations.

The obtained Pearson coefficients indicated an intermediate correlation between the overlap-volumes of the OAR and the treatment plan quality measured by the penalty score S. Within this patient collective, accumulated violations of relevant dosimetric criteria per single treatment plan showed a comparable correlation of large overlaps of both OAR with the PTV (maximum difference of 0.06 Pearson’s correlation between bladder and rectum).

In addition, more than two thirds (IGRT) and at least 86% (re-optimization approaches) of treatment plans with OAR overdoses could be predicted by V_PTV ∩ bladder_ and V_PTV ∩ rectum_. Consequently, overlap-volumes could serve as a fast and reliable indicator for triggering treatment plan adaptation. As recently reported, ad-hoc offline re-planning still constitutes the major part of all performed ART techniques for prostate cancer [48]. Pre-defined action levels based on simple structure metrics and different WSO approaches thus could make a difference in the transition process from offline to online ART treatments.

While deformable image registration (DIR)-based sCT generation includes a potential deformation of the CBCT anatomy based on the matching to the reference pCT [49], the characteristic feature of the presented neural network-based sCT generation is that the geometry remains identical to the CBCT and solely Hounsfield units or, respectively, electron densities are changed by the sCT algorithm. Different outcomes of image registration between sCT or CBCT and the pCT are negligibly small since a preliminary comparison of all treatment plans between the IGRT approach and the approach having an ISO defined as the center-of-mass of the target volume on the sCT merely changed the mean D_98%_(PTV) parameter from 32.9 to 33.3 Gy. For DIR-based sCT generation the sCT should be generated prior to image registration to minimize errors.

With regard to clinical feasibility in an online treatment workflow, the lack of tracking devices such as markers/transponder beacons [50], ultrasound [51] or EPID megavoltage imaging [52] constitute a shortcoming of the presented study since they are crucial for the monitoring of time-dependent intrafractional organ motion during the daily treatment plan delivery. All steps of an online adaptive workflow including image processing, segmentation and plan adaptation have to be kept as short as possible in order to avoid losing the benefits generated through this adaptation. Thus, the presented adaptive re-planning methods need to be combined with intrafractional monitoring devices or, for example, an acquisition of a second CBCT prior to the delivery of the adapted treatment plan for an eventual clinical implementation [35, 53, 54].

Despite the study’s retrospective setup, the presented adaptive planning approaches can ensure accurate daily dose delivery and contribute to the general motivation of performing daily online ART for prostate cancer radiotherapy [55]. Several offline and hybrid approaches already proved to be efficient for compensating organ movement but focused on a normo-fractionated treatment scheme with larger PTV margins, more than five fractions or solely on maintaining target coverage [3, 15, 56]. This study revealed the deficiencies of an IGRT approach and, more importantly, offered several adaptation techniques focusing on available planning time (ART1) and increasing target coverage (ART2) or reducing OAR dosage (ART3). Although there has been a long-time debate about the actual benefit of adaptive strategies with respect to their costs [57–59], promising results for daily treatment modifications have been obtained by fast and automated tools for image correction [10, 60], segmentation [61, 62] and treatment planning [63]. As previously reported, converting CBCT into sCT and subsequent image segmentation including manual correction of the generated structures takes up to 30 s, 30 s and 5.2 ± 1.6 min [10]. Together with the presented re-optimization approaches an end-to-end adaptive workflow can eventually become feasible within a reasonable timeframe of minimum 0.5 min + 0.5 min + 5.2 min + 2.6 min (ART1 approach) = 8.8 min, being in line with reported adaptation times of 10 min including online plan QA [64].

## Conclusion

This study demonstrated that the three adaptation approaches were able to restore and achieve the dosimetric goals of a prostate SBRT protocol and thus substantially improved treatment plan penalty in comparison to the conventional IGRT approach. Besides standard dose-volume metrics, a penalty score and overlap-volumes could identify the differences of dosimetric benefits among three different adaptation approaches, facilitating the decision when to apply which adaptation strategy.

## Data Availability

The datasets analyzed in the presented study are available from the corresponding author on reasonable request.

## Abbreviations

ART: Adaptive radiotherapy
CT: Computed tomography
CBCT: Cone-beam CT
CTV: Clinical target volume
Cycle-GAN: Cycle-generative adversarial network
DIR: Deformable image registration
DVH: Dose volume histogram
EPID: Electronic portal imaging device
IGRT: Image-guided radiotherapy
IQR: Inter-quartile range
ISO: Isocenter
OAR: Organs at risk
PACE: Prostate advances in comparative evidence
pCT: Planning CT
PTV: Planning target volume
SAM: Segment aperture morphing
SBRT: Stereotactic body radiotherapy
sCT: Synthetic CT
SD: Standard deviation
SV: Seminal vesicles
VMAT: Volumetric modulated arc therapy
WSO: Warm start optimization
XVI: X-ray volumetric imaging
